# Supplementary material for: Variations in the Sensory Attributes of Infant Formula among Batches and Their Impact on Maternal Consumer Preferences: A Study Combining Consumer Preferences, Pivot Profile, and Quantitative Descriptive Analysis
Source: Foods. 2024 Sep 7;13(17):2839. doi: 10.3390/foods13172839 (PMC11394814; doi:10.3390/foods13172839)
Supplement: Supplementary file 1 [file foods-13-02839-s001.zip › foods-3161214-supplementary.pdf]

Supplementary Materials

Table S1. Odor and flavor intensity referencing scales.

	Intensity level								
9-point scale	1	2	3	4	5	6	7	8	9
n-butanol (μg/L)	20	40	80	160	320	640	1280	2560	5120
sugar (g/L)	0.25	0.5	1	2	4	8	16	32	64

Table S2. Positive frequencies and negative frequencies for various attributes of the 12 batches of one-stage IFs from Pivot Profile experiment (based on the responses of all the 100 consumers).

Attribute		A14	A11	A10	A9	A8	A7	A6	A5	A4	A3	A2	A1
Milky	+*	29	13	16	19	20	28	18	17	25	27	35	30
	-*	30	46	47	51	41	34	38	38	37	28	32	30
Creamy	+	5	12	11	11	9	9	9	17	12	12	12	16
	-	10	10	13	14	5	12	12	11	4	7	11	16
Milk candy	+	6	6	1	5	7	6	4	3	6	5	7	8
	-	7	8	9	12	8	11	9	8	3	6	7	9
Fishy	+	17	66	29	36	19	8	24	36	36	11	3	9
	-	3	4	2	4	5	8	4	7	2	6	3	12
Cereal	+	10	5	8	7	5	7	6	4	7	2	3	5
	-	0	1	2	3	3	1	1	0	0	3	3	4
Eggy	+	10	16	20	24	12	8	9	10	12	8	4	12
	-	0	2	4	4	1	3	5	7	1	5	6	7
Soy milk	+	15	9	25	17	15	12	15	10	14	10	10	7
	-	4	6	5	9	2	4	6	5	3	5	5	7

Sweet	+	13	4	5	5	10	11	7	10	9	9	7	9
	-	8	11	10	14	7	12	15	15	9	9	5	13
Oxidation	+	3	12	4	6	4	1	2	5	2	3	1	1
	-	0	0	0	0	0	0	0	0	0	0	2	0
T-sweet	+	18	7	9	14	17	18	14	13	17	15	20	18
	-	25	31	37	31	24	27	43	32	24	24	23	28
T-Salty	+	2	9	9	17	4	2	5	4	4	2	5	8
	-	2	3	3	2	3	1	2	3	1	2	3	5
T-mellow	+	12	11	10	12	15	15	11	16	8	14	6	19
	-	16	24	22	18	17	30	30	26	17	22	18	24

*+：Pivot Profile Check the total frequency of " more than the pivot " in the experiment; -: Pivot Profile Check the total frequency of " less than the pivot " in the experiment.

Table S3. Positive frequencies and negative frequencies for various attributes of the 12 batches of one-stage IFs from Pivot Profile experiment (based on the responses of the 62 consumers of cluster 1 in AHC analysis).

Attribute		A14	A11	A10	A9	A8	A7	A6	A5	A4	A3	A2	A1
Milky	+*	15	6	9	9	15	16	9	8	20	16	26	15
	-*	17	25	29	32	19	21	19	23	18	15	15	20
Creamy	+	3	8	8	9	7	6	4	11	9	6	10	12
	-	8	7	9	10	2	10	7	6	3	1	6	11
Milk candy	+	3	2	1	4	6	3	3	3	6	3	6	6
	-	4	5	5	7	3	9	3	5	1	5	3	7
Fishy	+	8	43	17	23	8	6	16	21	19	5	8	7
	-	2	2	1	3	2	4	2	6	1	5	2	7
Cereal	+	7	1	6	6	2	3	4	2	4	2	2	1
	-	0	1	1	2	1	1	1	0	0	3	1	1
Eggy	+	5	11	10	13	9	6	5	7	8	5	2	7
	-	0	1	3	2	1	1	3	5	0	4	3	6
Soy milk	+	14	5	19	13	12	8	11	7	7	6	7	7
	-	2	5	5	8	1	3	5	3	2	4	4	3

Sweet	+	5	1	3	3	9	9	4	5	8	4	7	5
	-	5	9	7	11	5	6	9	10	4	5	2	12
Oxidation	+	2	8	2	3	0	1	0	4	1	3	0	1
	-	0	0	0	0	0	0	0	0	0	0	1	0
T-sweet	+	11	3	6	10	7	10	7	7	10	11	12	8
	-	12	20	19	16	12	12	25	17	12	12	9	13
T-Salty	+	1	7	5	9	3	2	2	1	1	1	3	5
	-	1	1	1	1	2	0	2	3	0	0	1	2
T-mellow	+	3	6	3	4	11	9	8	8	5	7	4	9
	-	10	12	17	14	7	18	17	14	4	12	7	15

*+: Pivot Profile Check the total frequency of " more than the pivot " in the experiment; -: Pivot Profile Check the total frequency of " less than the pivot " in the experiment.

Table S4. Positive frequencies and negative frequencies for various attributes of the 12 batches of one-stage IFs from Pivot Profile experiment (based on the responses from of 38 of cluster 2 in AHC analysis).

Attribute		A14	A11	A10	A9	A8	A7	A6	A5	A4	A3	A2	A1
Milky	+*	15	7	5	10	5	12	8	9	4	11	9	15
	-*	13	21	19	19	21	13	19	16	20	13	17	10
Creamy	+	2	4	3	6	3	3	5	6	3	6	2	4
	-	2	3	4	4	3	2	5	5	1	6	5	5
Milk candy	+	3	4	0	1	1	3	1	0	0	2	1	2
	-	3	3	4	5	5	2	6	3	2	1	4	2
Fishy	+	9	23	12	13	11	2	8	16	17	6	3	2
	-	1	2	1	1	3	4	2	1	1	1	1	5
Cereal	+	3	4	2	1	3	4	2	2	3	0	1	4
	-	1	1	0	1	2	0	0	0	0	0	2	3
Eggy	+	5	5	10	11	3	2	4	3	3	3	2	5
	-	0	1	1	2	0	2	2	2	1	1	3	1
Soy milk	+	1	4	6	4	2	4	4	3	7	4	3	0
	-	2	1	0	1	1	1	3	1	1	1	1	4

Sweet	+	8	3	2	2	1	2	3	5	1	5	0	4
	-	3	2	3	3	2	6	6	5	5	4	3	1
Oxidation	+	1	4	2	3	4	0	2	1	1	0	1	0
	-	0	0	0	0	0	0	0	0	0	0	1	0
T-sweet	+	7	4	3	4	12	8	7	6	8	4	8	10
	-	13	11	18	15	11	15	18	16	12	12	14	15
T-Salty	+	1	2	4	8	2	0	3	5	3	1	2	3
	-	1	2	2	1	1	1	0	0	1	2	2	3
T-mellow	+	9	5	7	8	3	6	3	8	3	7	2	10
	-	6	12	5	4	8	12	13	12	14	10	11	9

*+: Pivot Profile Check the total frequency of " more than the pivot " in the experiment; -: Pivot Profile Check the total frequency of " less than the pivot " in the experiment.

Table S5. Positive frequencies and negative frequencies for various attributes of the 12 batches of three-stage IFs from Pivot Profile experiment (based on the responses of all the 100 consumers).

Attribute		C14	C11	C10	C9	C8	C7	C6	C5	C4	C3	C2	C1
Milky	+*	10	8	10	10	8	8	15	13	16	19	25	17
	-*	42	38	42	37	49	44	38	30	31	36	30	26
Creamy	+	16	10	13	10	14	12	12	14	11	11	9	13
	-	6	9	7	9	9	5	6	3	5	5	5	3
Milk candy	+	6	6	7	12	4	2	6	12	13	5	4	11
	-	11	3	8	9	5	10	16	6	4	8	9	6
Fishy	+	37	64	74	36	41	40	76	23	28	7	9	13
	-	5	2	1	3	3	3	3	4	5	5	3	3
Cereal	+	5	3	6	5	13	5	4	4	4	3	6	5
	-	2	3	1	0	0	2	4	5	1	1	2	2
Eggy	+	21	21	20	18	10	12	22	16	9	5	6	14
	-	4	2	4	10	3	1	1	4	3	7	2	0
Soy milk	+	12	6	9	10	13	6	10	4	14	10	14	15
	-	7	6	4	5	7	4	3	10	9	5	4	3

Sweet	+	6	3	5	6	5	5	4	10	7	8	5	7
	-	11	6	10	4	10	13	8	6	6	7	5	11
Oxidation	+	5	10	10	6	8	6	8	3	4	0	3	2
	-	1	1	0	0	0	0	0	2	0	1	0	1
T-sweet	+	5	4	17	20	15	12	14	20	12	15	19	23
	-	34	40	36	28	30	36	25	23	25	30	30	34
T-Salty	+	14	16	9	11	10	10	16	5	7	7	4	7
	-	2	1	5	2	2	2	4	7	4	2	4	6
T-mellow	+	4	3	10	5	5	4	4	13	10	14	9	9
	-	20	23	21	25	13	21	22	19	13	19	17	21

*+: Pivot Profile Check the total frequency of " more than the pivot " in the experiment; -: Pivot Profile Check the total frequency of " less than the pivot " in the experiment.

Table S6. Positive frequencies and negative frequencies for various attributes of the 12 batches of three-stage IFs from Pivot Profile experiment (based on the responses of the 44 consumers of cluster 3 in AHC analysis).

Attribute		C14	C11	C10	C9	C8	C7	C6	C5	C4	C3	C2	C1
Milky	+*	5	5	8	6	4	3	10	8	10	6	10	9
	-*	20	10	14	16	18	20	15	9	14	18	14	6
Creamy	+	7	7	7	4	8	5	5	7	5	4	7	9
	-	2	4	2	5	4	4	2	0	2	4	3	1
Milk candy	+	4	5	6	7	3	1	3	6	5	2	3	7
	-	3	1	3	4	2	5	7	5	2	4	3	2
Fishy	+	17	17	26	17	11	14	26	6	8	3	5	6
	-	4	2	1	3	2	2	2	3	2	3	3	1
Cereal	+	1	2	4	3	7	0	3	2	1	1	3	4
	-	1	2	0	0	0	2	0	4	0	1	0	2
Eggy	+	9	6	7	5	5	4	11	8	3	3	2	6
	-	2	2	3	5	0	1	1	2	2	3	2	2
Soy milk	+	7	4	3	4	8	5	6	2	6	7	9	7
	-	1	3	2	1	3	1	1	5	5	1	2	2

Sweet	+	4	3	3	1	3	3	2	4	5	3	1	3
	-	3	2	4	3	7	6	3	4	2	2	2	6
Oxidation	+	1	1	1	4	1	1	0	0	0	0	0	1
	-	1	1	0	0	0	0	1	0	1	1	0	1
T-sweet	+	1	4	10	5	11	5	9	9	7	9	6	10
	-	11	15	11	14	11	17	9	11	10	14	17	16
T-Salty	+	7	4	5	5	3	5	8	3	1	4	4	5
	-	1	0	2	1	1	0	2	5	2	1	3	3
T-mellow	+	3	2	9	2	2	2	3	8	5	4	3	5
	-	12	11	10	14	4	11	11	5	7	9	9	13

*+: Pivot Profile Check the total frequency of " more than the pivot " in the experiment; -: Pivot Profile Check the total frequency of " less than the pivot " in the experiment.

Table S7. Positive frequencies and negative frequencies for various attributes of the 12 batches of three-stage IFs from Pivot Profile experiment (based on the responses of the 56 consumers of cluster 4 in AHC analysis).

Attribute		C14	C11	C10	C9	C8	C7	C6	C5	C4	C3	C2	C1
Milky	+*	5	3	2	4	4	5	5	5	6	13	15	7
	-*	22	28	28	21	31	24	23	19	17	18	16	19
Creamy	+	9	3	6	6	6	7	7	6	6	7	2	4
	-	4	5	5	4	5	1	4	3	3	1	2	2
Milk candy	+	2	1	1	5	1	1	3	6	8	3	1	4
	-	8	2	5	5	3	5	9	1	2	4	6	4
Fishy	+	20	47	48	19	30	26	50	15	20	4	4	6
	-	1	0	0	0	1	1	1	1	3	2	0	2
Cereal	+	4	1	2	2	6	5	1	2	3	2	3	1
	-	1	1	1	0	0	0	4	1	1	0	2	0
Eggy	+	12	15	13	13	5	8	11	8	6	2	4	8
	-	2	0	1	3	8	0	0	2	1	4	2	0
Soy milk	+	5	2	6	6	5	1	4	2	8	3	5	8
	-	6	3	2	5	4	3	2	5	4	4	2	1

Sweet	+	4	0	7	15	4	7	5	10	5	6	15	12
	-	8	4	0	6	3	7	5	2	4	5	3	2
Oxidation	+	4	9	9	2	7	5	7	3	4	0	3	1
	-	0	0	0	0	0	0	0	1	0	0	0	0
T-sweet	+	4	0	7	15	4	7	5	10	5	6	13	12
	-	23	25	25	13	19	19	16	11	15	16	13	18
T-Salty	+	7	12	5	6	7	5	8	1	6	3	0	2
	-	1	1	3	0	1	2	2	2	2	1	1	3
T-mellow	+	1	1	1	3	3	2	1	5	5	10	6	4
	-	8	12	11	12	9	10	11	12	6	10	8	8

*+: Pivot Profile Check the total frequency of " more than the pivot " in the experiment; -: Pivot Profile Check the total frequency of " less than the pivot " in the experiment.

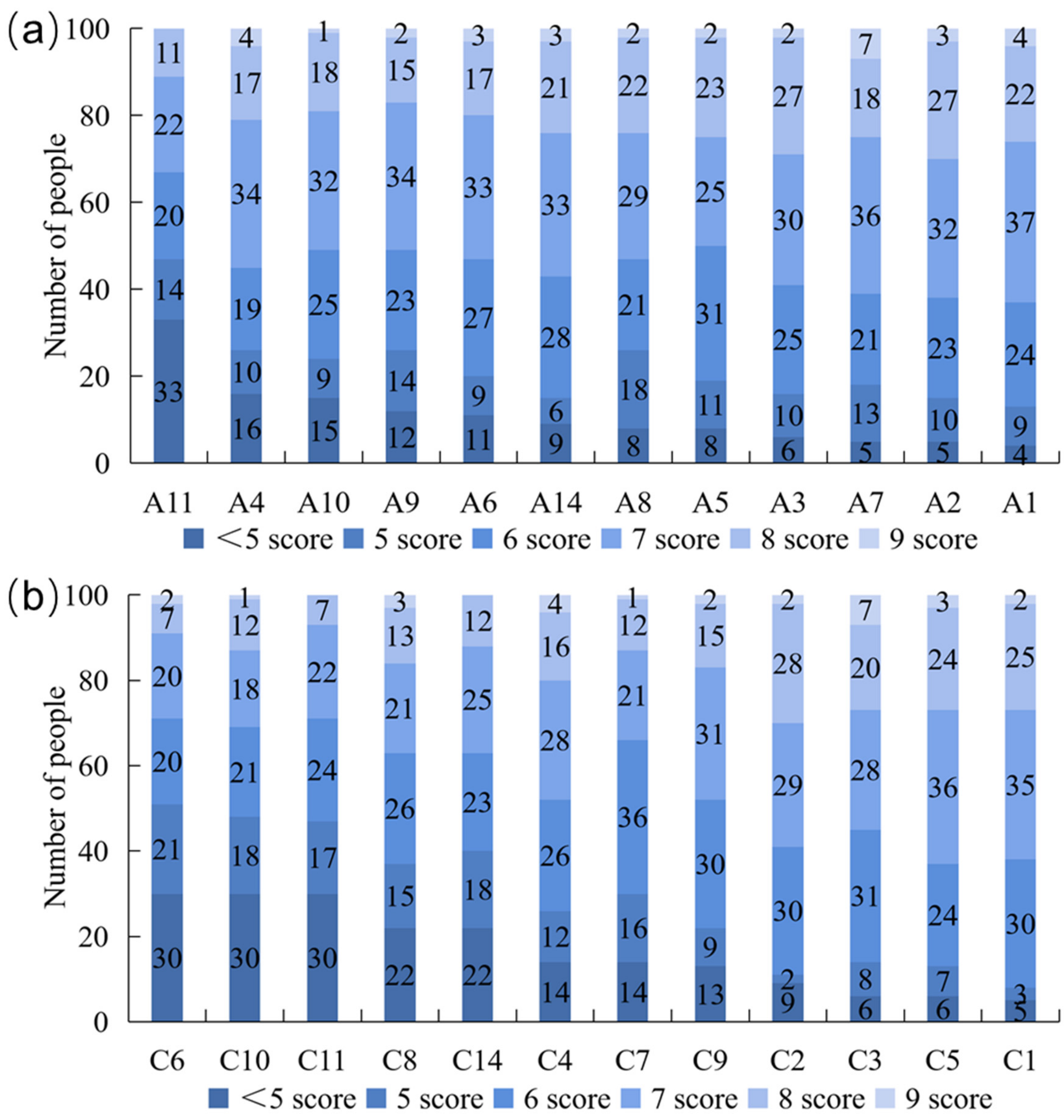

Figure S1. Stacked bars of preference ratings for (**a**) one-stage IFs and (**b**) three-stage IFs from 100 consumers.
